# Behavioural responses of brown bears to roads and hunting disturbance

**DOI:** 10.1002/ece3.11532

**Published:** 2024-06-14

**Authors:** Ludovick Brown, Andreas Zedrosser, Jonas Kindberg, Fanie Pelletier

**Affiliations:** ^1^ Département de Biologie Université de Sherbrooke Sherbrooke Canada; ^2^ Department of Natural Sciences and Environmental Health University of South‐Eastern Norway Bø in Telemark Norway; ^3^ Institute for Wildlife Biology and Game Management University for Natural Resources and Life Sciences Vienna Austria; ^4^ Norwegian Institute for Nature Research Trondheim Norway; ^5^ Department of Wildlife, Fish and Environmental Studies Swedish University of Agricultural Sciences Umeå Sweden

**Keywords:** disturbance, hunting, landscape of fear, movement rate, risk perception, *Ursus arctos*

## Abstract

Harvest regulations commonly attenuate the consequences of hunting on specific segments of a population. However, regulations may not protect individuals from non‐lethal effects of hunting and their consequences remain poorly understood. In this study, we compared the movement rates of Scandinavian brown bears (*Ursus arctos*, *n* = 47) across spatiotemporal variations in risk in relation to the onset of bear hunting. We tested two alternative hypotheses based on whether behavioural responses to hunting involve hiding or escaping. If bears try to reduce risk exposure by avoiding being detected by hunters, we expect individuals from all demographic groups to reduce their movement rate during the hunting season. On the other hand, if bears avoid hunters by escaping, we expect them to increase their movement rate in order to leave high‐risk areas faster. We found an increased movement rate in females accompanied by dependent offspring during the morning hours of the bear hunting season, a general decrease in movement rate in adult lone females, and no changes in males and subadult females. The increased movement rate that we observed in females with dependant offspring during the hunting season was likely an antipredator response because it only occurred in areas located closer to roads, whereas the decreased movement rate in lone females could be either part of seasonal activity patterns or be associated with an increased selection for better concealment. Our study suggests that female brown bears accompanied by offspring likely move faster in high‐risk areas to minimize risk exposure as well as the costly trade‐offs (i.e. time spent foraging vs. time spent hiding) typically associated with anti‐predator tactics that involve changes in resource selection. Our study also highlights the importance of modelling fine‐scale spatiotemporal variations in risk to adequately capture the complexity in behavioural responses caused by human activities in wildlife.

## Graphical abstract

In this study, we investigated the movement behaviour of brown bears in response to roads and human disturbances by using linear mixed‐effect models. We found that females with dependant offspring increase their movement speed when located close to roads during the bear hunting season.

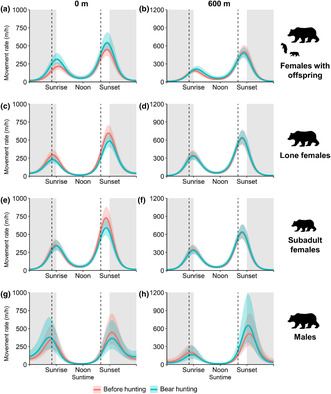

## 1 INTRODUCTION

Recreational hunting has well‐documented consequences on wildlife, including behavioural changes, reduction in body size in the exploited segments of populations, altered population structure and increased mortality rates (Darimont et al., 2009, Fenberg & Roy, 2008, Leclerc et al., 2017). Humans are generally perceived as a super predator (Darimont et al., 2015) and animals often respond to this perceived risk by altering their behaviour to reduce their exposure to this risk (Gaynor et al., 2018). These behavioural responses have also been reported when human activities pose little to no risk for wild animals (Gaynor et al., 2018; Rode et al., 2007).

The spatiotemporal variations in perceived predation risk, commonly referred to as landscape of fear (Brown & Kotler, 2004; Gaynor et al., 2019; Laundré et al., 2001), may induce different types of antipredator responses in wildlife. For instance, animals may shift to nocturnality to avoid encounters with humans (Gaynor et al., 2018). Other behavioural tactics that are commonly reported include avoiding high‐risk areas (Suraci et al., 2019) or initiating a flight response to escape predators (Cooper, 2009). The anti‐predator tactics that animals use are often dependent on external factors such as the environment or predator traits, which may ultimately influence how animals respond and how threats are perceived (Camp et al., 2012; Stankowich & Blumstein, 2005).

Antipredator behaviours can be energetically costly and generally imply a trade‐off with resource acquisition (Lima & Bednekoff, 1999; Lima & Dill, 1990). Thus, animals need to be able to detect spatiotemporal variation in actual risk to avoid over‐allocating time to antipredator behaviours when they are not required. For instance, perceived risks associated with human activities may lead animals to avoid areas that contain essential resources (Dwinnell et al., 2019; Hertel et al., 2016), which in turn may decrease foraging opportunities. Therefore, we can expect animals to allocate more antipredator behaviours at places and times with the highest perceived risks.

In this study, we used the brown bear (*Ursus arctos*) as a model species to evaluate how an intensively hunted species navigates small‐scale spatiotemporal variations in perceived risk. In Sweden, all brown bears can be harvested during the legal hunting period, except members of family groups (i.e. females accompanied by dependent offspring of any age), which are legally protected (Van de Walle et al., 2018). Swedish hunters are generally law abiding and only very few bears from family groups are killed during the hunting season. However, hunters in Sweden commonly use baying dogs to trail and drive bears (Bischof et al., 2008), which also results in pursuits of family groups because dogs do not discriminate between lone bears and family groups. Therefore, we expect a similar behavioural response to hunting in unprotected bears and family groups because they can also be tracked and chased by dogs, although they would not be shot. Previous work has shown that brown bears respond to hourly variation in hunting risk by reducing foraging activities and movements during the morning hours when the risk of hunter‐caused mortality is highest (Hertel et al., 2016; Ordiz et al., 2012); however, Brown et al. (2023) have shown that female brown bears move faster when travelling through high‐risk areas to presumably reduce risk exposure. Therefore, moving faster to escape hunters may be an alternative antipredator tactic, especially considering that Leclerc et al. (2019) have shown that hunters in Sweden are more likely to shoot bears that move more slowly. Here, we build on these previous studies and investigate if the movement rates and activity patterns of brown bears from different demographic groups change across spatiotemporal variations in perceived predation risk.

Several studies have shown that predation risk from hunters is heterogeneously distributed across space and time, but it is also highly predictable because it is restricted to legal hunting hours during daytime and generally occurs in areas close to roads (Gaynor et al., 2022; Perry et al., 2020; Steyaert et al., 2016). We first hypothesized that brown bears change their behaviour after the onset of the hunting season in areas used by hunters to reduce the risk of detection. As hunting activities mainly occur near roads, we expect bears to decrease their movement rate when located near roads during legal hunting hours, especially during the morning when most hunting‐caused mortalities occur (Hertel et al., 2016). Alternatively, we hypothesized that bears change their behaviour to escape hunters during legal hunting hours. We predict that brown bears increase their movement rate to leave high‐risk areas faster during the morning.

## 2 METHOD

### 2.1 Study area

Our study area was located in south‐central Sweden (~61° N, 15° E) during 2016–2019. The landscape contains a highly managed boreal forest with a dense network of forest roads and low human density (Martin et al., 2010; Ordiz et al., 2013). Bear hunting in Sweden starts on August 21 and lasts until October 15 or until the regional quota has been filled, whichever comes first (Bischof et al., 2008). Bears are almost exclusively hunted by baying dogs that pick up scent trails and track bears with hunters trying to catch up or intercept their trajectory (Bischof et al., 2008; Leclerc et al., 2019). All bears can be legally harvested except members of family groups, which are protected year‐round (Van de Walle et al., 2018).

### 2.2 Bear capture and telemetry

We collected data on 47 brown bears (*n* = 92 bear‐years) from four different demographic groups [i.e. females with dependent offspring, *n* = 12 individuals, *n* = 19 bear‐years; lone females, *n* = 21 individuals, *n* = 32 bear‐years; subadult females (<4 years old), *n* = 21 individuals, *n* = 32 bear‐years; males, *n* = 8 individuals, *n* = 9 bear‐years] that survived the hunting season. No individuals were monitored for more than 4 years, and some individuals occur in more than one group (e.g. a subadult becoming an adult). Animals were darted from a helicopter with a remote drug delivery system (Dan‐Inject, Børkop, Denmark) and equipped with GPS‐GMS collars (GPS Plus; Vectronic Aerospace, Berlin, Germany) that were programmed with a 1 h fix rate. In this study, we considered subadult and adult males as a single group due to the low sample size. See Arnemo and Evans (2017) for more details about the capture protocol. All capture and handling protocols were approved by the Swedish Ethical Committee on Animal Research, Uppsala (C18/15), and the Swedish Environmental Protection Agency (NV‐00741‐18, NV‐01758‐14).

### 2.3 Data handling

We only used relocation data with dilution of precision < 10 (D'Eon & Delparte, 2005). Our final data set contained 39,559 GPS locations from 92 bear‐years collected at 1 h intervals between 10 and 31 August, during 2016–2019. We defined the period ‘before hunting’ as the 11 days (10–20 August) before the onset of the bear hunt and used 31 August as a cut‐off for the ‘bear hunt’ (21–31 August) to avoid any interference with the onset of moose hunting on the first Monday of September. GPS locations were converted to animal tracks with the package *amt* (Signer et al., 2019), which calculates the distance travelled in 1 h as a straight line between consecutive locations. The distance to the closest road (in meters) was extracted from a distance raster (10 m resolution) based on the National Road Data Base of the Swedish Transport Administration (Trafikverket, https://www.trafikverket.se).

### 2.4 Statistical analyses

We modelled the distance travelled between two consecutive relocations (i.e. movement rate in m/h) in bears with linear mixed‐effect models in the *nlme* package (Pinheiro et al., 2021), which allowed us to include a *corCAR1* structure and account for temporal autocorrelation. The model included the distance to the closest road as a proxy for risk, season (i.e. *before hunting* vs. *bear hunting*) and time of day. We converted clock time into solar time (hereafter referred to as sun time) in radians with sunrise and sunset standardized at π/2 and 3π/2, respectively (*overlap* package; (Ridout & Linkie, 2009)). We used the structure described in Richter et al. (2020) to model the effect of sun time with trigonometric functions [i.e. (sin(sun time) + cos(sun time) + 2∙sin(sun time) +2∙cos(sun time))]. We added nine two‐way interactions and four three‐way interactions between the variables distance to road, sun time and period. We used separate models for each demographic group to facilitate the interpretation of the results. Subadult and adult males were pooled into a single group due to the low sample size. We also added random slopes for sun time with bear‐year as a random intercept, thereby accounting for interindividual behavioural differences. We removed random slopes from the male model due to convergence issues and only kept the random intercept for bear‐year. We used diagnostic plots to ensure there were no major deviations from model assumptions (normality and homoscedasticity of residuals; Figure S1). The response variable was log‐transformed to achieve normality of the residuals and we added 1 to all observations to facilitate log‐transformation of steps with no movement (i.e. 0 m). The distance to the closest road was standardized to facilitate model convergence. We set α at 0.05 and all statistical analyses were carried out in R 4.1.0 (R Core Team, 2021).

## 3 RESULTS

**FIGURE 1:**
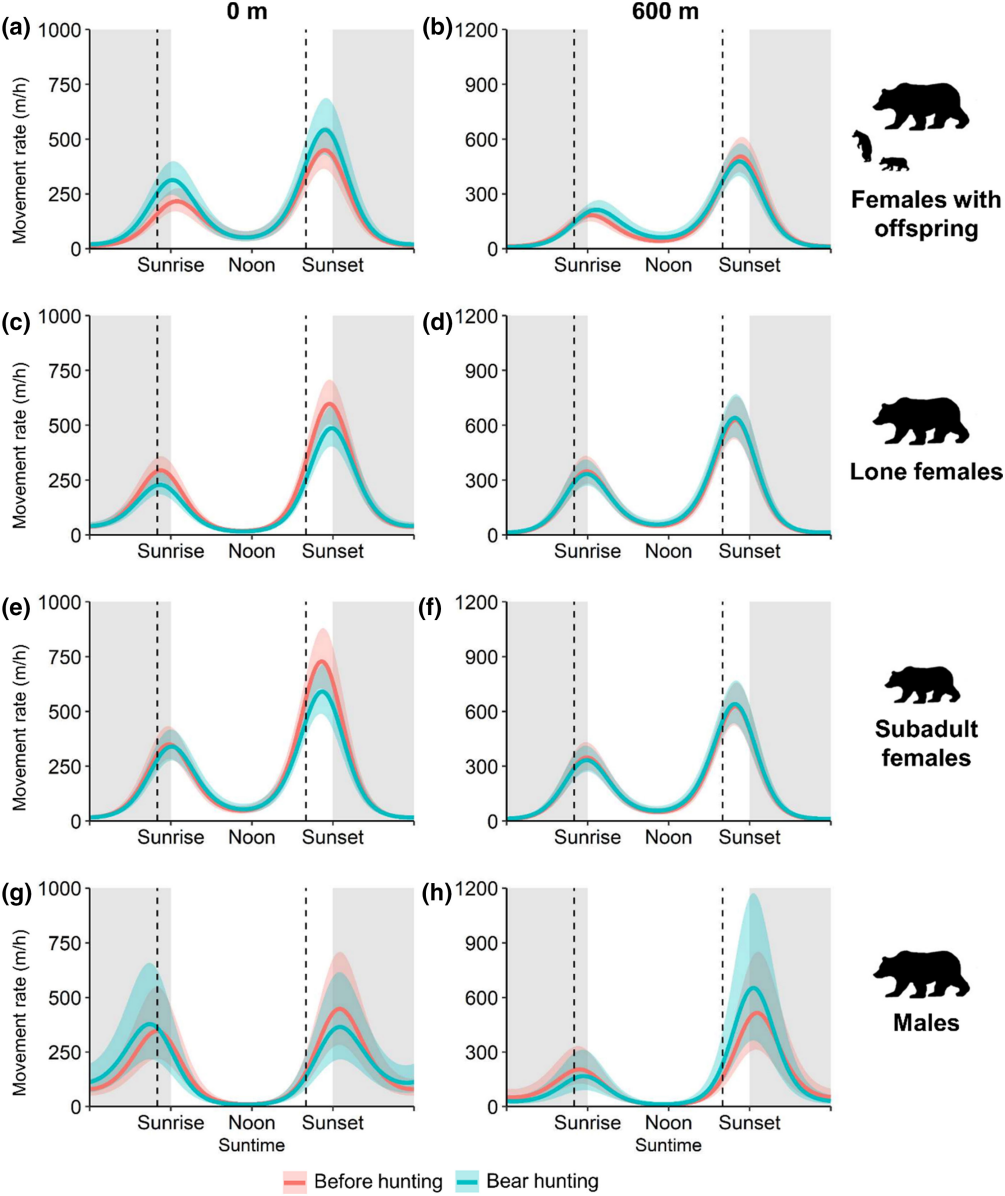
Predicted movement rate (m/h) with 95% confidence intervals for brown bears according to time of day during each season (*before hunting* and *bear hunting*) for females with dependent offspring (*n* = 19 bear‐years), lone females (*n* = 32 bear‐years), subadult females (*n* = 32 bear‐years) and males (*n* = 9 bear‐years) in south‐central Sweden, during 2016–2019. Movement rate was predicted at 0 and 600 m from the closest road and during each season (red = *before hunting season*, blue = *bear hunting season*). The vertical dashed lines show the start (left) and end (right) of legal hunting hours. Shaded areas represent nighttime, whereas the white areas represent daytime.

Bear movement rates followed a typical bimodal pattern with peaks around sunrise and sunset in all demographic groups (Figure 1). The movement rate of females with dependent offspring increased around the start of legal hunting hours during bear hunting in areas close to roads (Figure 1a), but this effect dissipated with increasing distance from roads (Figure 1b). Males exhibited activity patterns that were more nocturnal compared to females. The movement rate of females with dependent offspring generally increased during the bear hunting season (*β*_hunting_ = 0.12, *p* = .01; Table S1), whereas it decreased in lone females (*β*_hunting_ = −0.15, *p* < 0.001; Table S1) and remained the same in subadult females (*β*_hunting_ = 0.02, *p* = .56; Table S1) and males (*β*_hunting_ = −0.05, *p* = .69, Table S1). Other coefficient estimates are presented in Table S1.

**FIGURE 2:**
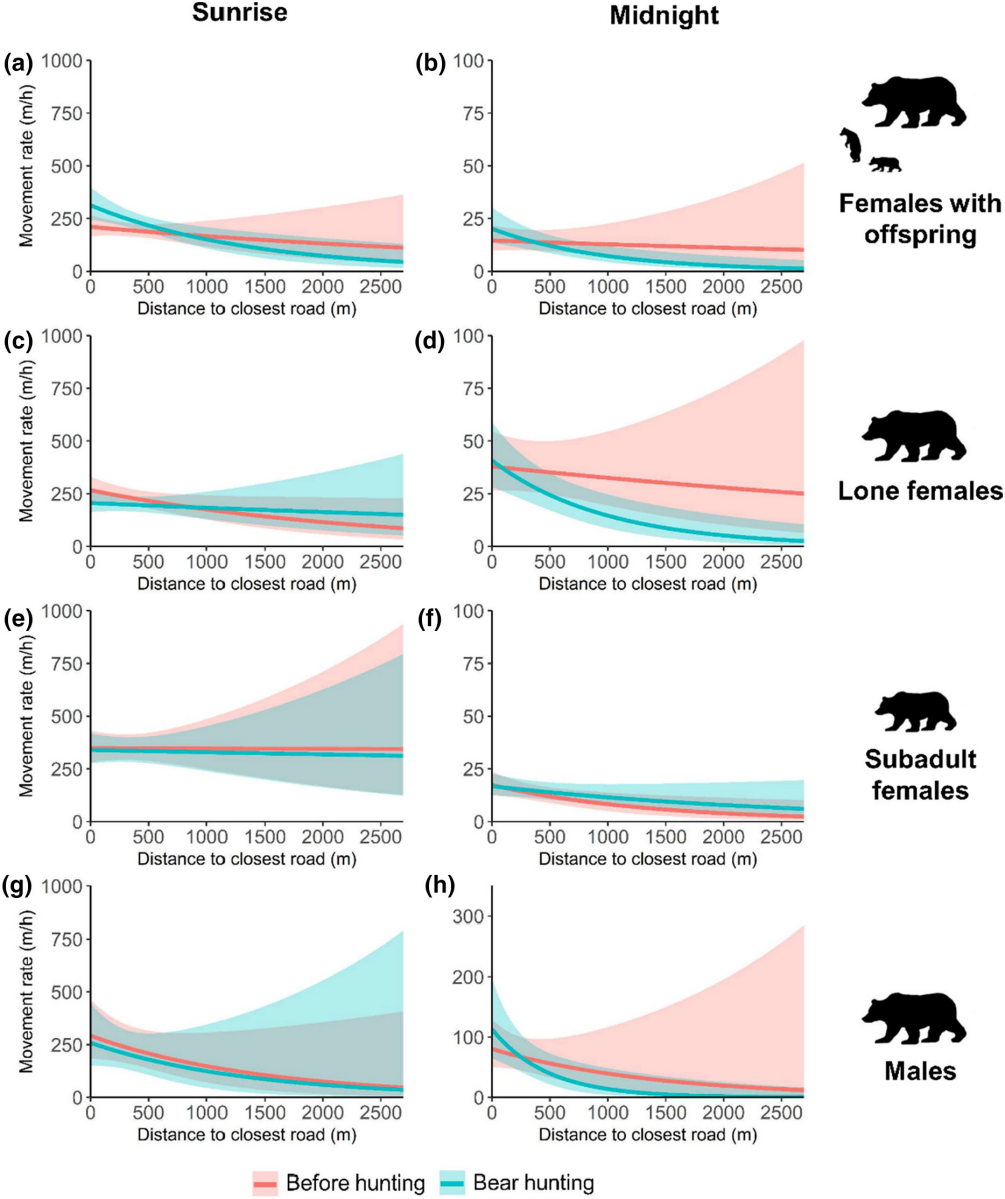
Predicted movement rate (m/h) with 95% confidence intervals for brown bears according to distance to the closest road for females with dependent offspring (*n* = 19 bear‐years), lone females (*n* = 32 bear‐years), subadult females (*n* = 32 bear‐years) and males (*n* = 9 bear‐years) in south‐central Sweden, during 2016–2019. Movement rate was predicted at sunrise (left panels) and midnight (right panels) from the closest road and during each season (red = *before hunting season*, blue = *bear hunting season*). Note the scale difference in the panels.

At sunrise, the period with the highest hunting risk, females with dependant offspring moved faster when closer to roads during bear hunting (Figure 2a). Lone females moved faster when closer to roads before the hunting period (Figure 2c), whereas roads had no effect on the movement rate of subadults at sunrise (Figure 2e). At midnight, the period with the lowest hunting risk, the effect of the distance to the closest road on movement rate was similar during bear hunting and before hunting for all groups (Figure 2).

## 4 DISCUSSION

We only found partial support for our prediction that bears change their movement rates in order to reduce the risk of detection, which was suggested by a reduced movement rate in solitary females during the hunting season. We also found partial support for our alternative hypothesis that bears change their movement rate to escape hunters. This hypothesis was supported by an increased movement rate in areas close to roads during the bear hunt in females with dependent offspring, which also suggests that legal protection from harvest did not influence their movement response in relation to the perceived threat from hunters. In contrast to our hypotheses and predictions, we found no effects of hunting on the movement rates of males and subadult females.

Our results suggest that the increased movement rate during the bear hunt in females with dependent offspring was likely an anti‐predator response because it was more pronounced closer to roads, where most bear mortalities occur in our study area (Steyaert et al., 2016). Members of family groups are protected and cannot be legally harvested by hunters in Sweden; however, they are still chased by hunting dogs that have been released to search and follow scent trails from bears. The presence of hunting dogs near roads, whether or not they engage in a pursuit, should still be perceived as a threat by females for their dependant offspring, resulting in an increased movement rate when travelling in high‐risk areas during the hunting season. Increased movement rate in response to hunting has been reported in moose (*Alces alces*) from Alaska, USA, where individuals inhabiting areas accessible for hunters have higher movement rates during the hunting season compared to individuals located in areas with poor road access (Brown et al., 2018). A similar pattern has been reported in mule deer (*Odocoileus hemionus*) from Oregon, USA, which also increased movement rates during the hunting season (Brown et al., 2020). In line with this trend, moose from Sweden generally move faster when located within 125 m of a road (Neumann et al., 2013), which suggests that increasing movement rate may minimize the time spent in high‐risk areas, thereby reducing risk exposure.

Increasing the selection of concealment is another antipredator response that is commonly reported in multiple species, including bears (Marantz et al., 2016; Ordiz et al., 2012; Paton et al., 2017); however, this tactic needs to be traded off with the time spent foraging (Lima & Dill, 1990). Females with dependent offspring may not be able to afford to forego due to higher energy requirements and may travel faster in areas with high perceived risk (i.e. close to roads) instead of altering resource selection and hiding when a threat is perceived. Brown et al. (2023) have shown previously that lone female brown bears in Sweden increase the selection for better concealment during the hunting season. This tactic would be consistent with the decreased movement rate that we observed in lone females and would suggest that antipredator responses in bears depend on their reproductive status. Previous studies have shown that moose, white‐tailed deer (*O. virginianus*) and caribou (*Rangifer tarandus*) are more sensitive to perceived risk when accompanied by juveniles (Burton et al., 2022; Higdon et al., 2019; Viejou et al., 2018). By using camera trap, Burton et al. (2022) showed that ungulates accompanied by juveniles generated fewer photos per event, which suggests that they move faster when travelling through high‐risk areas. Our results suggest that this trend may not be limited to ungulates but also applies to hunted large carnivores, even if they benefit from legal protection from harvest.

Our results suggest further that bear hunting has no important effects on the movement rates of male and subadult female bears, which contrasts with the results for adult females. The lack of behavioural responses in males could be explained by a switch to a more nocturnal activity pattern compared to females before the onset of hunting. Nocturnal activity patterns are common antipredator behaviour (Gaynor et al., 2018), and this behaviour combined with selection for greater concealment during the day could explain the lack of movement response in this demographic group. However, this explanation remains speculative as we have little information on resource selection in male bears in our project because the monitoring has mainly focused on females. The lack of a strong response by subadult females could be attributed to their lack of experience and overall weaker responses to variations in risk, which is also supported by the minimal effect of hunting and roads on their movement rate.

Our results seem to contradict those of an earlier study that investigated the movement response to hunting in Scandinavian brown bears. Ordiz et al. (2012) reported that, among all demographic groups, females with dependant offspring showed the smallest variations in movement rates during the hunting season. Although we found an increased movement rate in females with dependant offspring after the onset of hunting, we only observed this behaviour change when they were located close to roads during the morning, but we did not find significant differences in movement rate after the onset of hunting when they were located farther away from roads. Thus, the more general patterns found here are in line with previous work (Ordiz et al., 2012).

Fine‐scale analyses and more sensitive variables allowed us to unravel the movement response to hunting in females with dependent offspring. For instance, we used solar time instead of clock time, which was found to be more meaningful to animals (Richter et al., 2020), and we also included the distance to the closest road as a proxy for small‐scale variations in risk. Predation risk is heterogeneously distributed in space and time (Gaynor et al., 2022) and antipredator responses in wildlife should closely follow its distribution (Lima & Bednekoff, 1999; Lima & Dill, 1990). Consequently, antipredator responses could only occur in specific areas or time of the day and not accounting for the scale at which they occur could preclude their detections in modelling efforts.

Despite our attempts to explore fine‐scale variations in movement rate in response to predation risk from hunters, our analyses still present significant limitations that should be considered when interpreting the results. For instance, the analyses of male behavioural responses were based on a relatively small sample size of 9 bear‐years (8 individuals in total), which limits our ability to extrapolate the results to the whole population. Additionally, adult bears are often more risk adverse than younger individuals (Lamb et al., 2020) and may therefore respond differently to human disturbance. Pooling adults and subadults into a single group in the analyses may have masked specific responses from each segment during the hunting season. Another potential limitation is that we do not know how many bears were chased by dogs, but ultimately avoided being shot (either due to the legal protection of family groups or because they escape hunters). These pursuits can momentarily increase the movement rate in wildlife, including bears (Græsli et al., 2020; Le Grand et al., 2019), and induce lasting behavioural changes during the next 2 or 3 days (Ordiz et al., 2013), thereby introducing bias in our analyses. Future studies should consider tracking the movement of hunters and dogs to more accurately model spatiotemporal variations in risk and to be able to determine which bears actually were disturbed.

## 5 CONCLUSION

In this study, we found that female brown bears with dependent offspring increased their movement rate in response to hunting, which contrasts with the response or lack of response observed in other demographic groups. We suggest that higher costs associated with raising offspring may trigger different antipredator responses in female bears. Our results also highlight the importance of modelling fine‐scale variations in risk to appropriately document the behavioural responses in wildlife and this modelling strategy allowed us to uncover an antipredator response that only occurred in areas with the highest perceived risk. Predation risk is not uniform in time and space (Gaynor et al., 2022) and this heterogeneity should be reflected in modelling efforts to avoid masking important behavioural responses in wildlife.

## AUTHOR CONTRIBUTIONS

**Ludovick Brown:** Conceptualization (equal); formal analysis (lead); investigation (equal); methodology (equal); writing – original draft (lead). **Andreas Zedrosser:** Conceptualization (equal); supervision (equal); writing – review and editing (equal). **Jonas Kindberg:** Funding acquisition (equal); project administration (lead); writing – review and editing (equal). **Fanie Pelletier:** Conceptualization (equal); funding acquisition (equal); supervision (equal); writing – review and editing (equal).

## CONFLICT OF INTEREST STATEMENT

None.

## Supporting information


Data S1.

Table S1.
Figure S1.

## Data Availability

The data used in statistical analyses can be accessed on dryad: DOI: 10.5061/dryad.1zcrjdg1f The codes used to replicate statistical analyses are provided as electronic Supporting Information.
