# Supplementary material for: Behavioural responses of brown bears to roads and hunting disturbance
Source: Ecol Evol. 2024 Jun 14;14(6):e11532. doi: 10.1002/ece3.11532 (PMC11176727; doi:10.1002/ece3.11532)
Supplement: Supplementary file 2 — Table S1. Figure S1. [file ECE3-14-e11532-s001.docx]

**Supplementary material for**: Behavioural responses of brown bears to roads and hunting disturbance

**Authors:** Ludovick Brown^1^, Andreas Zedrosser^2,3^, Jonas Kindberg^4,5^, Fanie Pelletier^1^

^1^Département de biologie, Université de Sherbrooke, Sherbrooke, Canada

^2^ Department of Natural Sciences and Environmental Health, University of South-Eastern Norway, Bø, Norway

^3^Institute for Wildlife Biology and Game Management, University for Natural Resources and Life Sciences, Vienna, Austria

^4^Department of Wildlife, Fish and Environmental Studies, Swedish University of Agricultural Sciences, Umeå, Sweden

^5^Norwegian Institute for Nature Research, Trondheim, Norway

Corresponding author: Ludovick Brown, Département de biologie, Université de Sherbrooke, Sherbrooke, Québec, J1K 2R1, Canada, [Ludovick.Brown@USherbrooke.ca](mailto:Ludovick.Brown@USherbrooke.ca)

**Table S1**. Output of linear mixed effect models used to predict the movement rate (log m/h) of females with dependent offspring (*n* = 19 bear-years), lone females (*n* = 32 bear-years), subadult females (*n* = 32 bear-years), and males (*n* = 9 bear-years) in south-central Sweden, before and during the bear hunt between 2016-2019. Statistically significant results (α ˂ 0.05) are highlighted in bold. The reference category for hunting variable is “*Before hunting*”.

|  | Coefficient | Std. Error | *t*-value | *p*-value |  |  |  |
| --- | --- | --- | --- | --- | --- | --- | --- |
| **Fixed effects** |  |  |  |  |  | **Random effects** |  |
|  |  |  |  |  |  |  |  |
| *Females with dependent offspring* |  |  |  |  |  |  |  |
| **Intercept** | **4.48** | **0.04** | **101.59** | **0.00** |  | Intercept | 0.14 |
| Road_D | -0.02 | 0.03 | -0.90 | 0.37 |  | sin(suntime) | 0.18 |
| **sin(suntime)** | **-0.41** | **0.06** | **-6.93** | **0.00** |  | cos(suntime) | 0.74 |
| **cos(suntime)** | **-0.64** | **0.18** | **-3.65** | **0.00** |  | sin(2*suntime) | 0.29 |
| sin(2*suntime) | 0.13 | 0.08 | 1.68 | 0.09 |  | cos(2*suntime) | 0.14 |
| **cos(2*suntime)** | **-1.20** | **0.05** | **-23.98** | **0.00** |  | Residuals | 1.41 |
| **huntingBear** | **0.12** | **0.04** | **2.68** | **0.01** |  |  |  |
| Road_D:sin(suntime) | -0.04 | 0.03 | -1.24 | 0.22 |  |  |  |
| Road_D:cos(suntime) | 0.01 | 0.04 | 0.28 | 0.78 |  |  |  |
| Road_D:sin(2*suntime) | 0.03 | 0.03 | 0.96 | 0.34 |  |  |  |
| Road_D:cos(2*suntime) | -0.02 | 0.03 | -0.49 | 0.63 |  |  |  |
| Road_D:huntingBear | -0.07 | 0.04 | -1.90 | 0.06 |  |  |  |
| sin(suntime):huntingBear | 0.09 | 0.06 | 1.53 | 0.13 |  |  |  |
| cos(suntime):huntingBear | -0.06 | 0.06 | -0.99 | 0.32 |  |  |  |
| sin(2*suntime):huntingBear | -0.01 | 0.06 | -0.22 | 0.82 |  |  |  |
| cos(2*suntime):huntingBear | -0.02 | 0.06 | -0.45 | 0.65 |  |  |  |
| Road_D:sin(suntime):huntingBear | -0.01 | 0.05 | -0.16 | 0.88 |  |  |  |
| **Road_D:cos(suntime):huntingBear** | **-0.16** | **0.05** | **-2.96** | **0.00** |  |  |  |
| Road_D:sin(2*suntime):huntingBear | -0.07 | 0.05 | -1.44 | 0.15 |  |  |  |
| Road_D:cos(2*suntime):huntingBear | 0.03 | 0.05 | 0.68 | 0.50 |  |  |  |
|  |  |  |  |  |  |  |  |
|  |  |  |  |  |  |  |  |
|  |  |  |  |  |  |  |  |
|  |  |  |  |  |  |  |  |
|  |  |  |  |  |  |  |  |
|  |  |  |  |  |  |  |  |
|  |  |  |  |  |  |  |  |
| **Table S1 (continued)** |  |  |  |  |  |  |  |
|  |  |  |  |  |  |  |  |
| *Lone females* |  |  |  |  |  |  |  |
| **Intercept** | **4.60** | **0.04** | **113.55** | **0.00** |  | Intercept | 0.17 |
| Road_D | -0.04 | 0.02 | -1.60 | 0.11 |  | sin(suntime) | 0.15 |
| **sin(suntime)** | **-0.41** | **0.05** | **-8.96** | **0.00** |  | cos(suntime) | 0.77 |
| **cos(suntime)** | 0.24 | 0.14 | 1.71 | 0.09 |  | sin(2*suntime) | 0.19 |
| **sin(2*suntime)** | **0.47** | **0.05** | **9.92** | **0.00** |  | cos(2*suntime) | 0.20 |
| **cos(2*suntime)** | **-1.26** | **0.05** | **-26.05** | **0.00** |  | Residuals | 1.47 |
| **huntingBear** | **-0.15** | **0.04** | **-3.86** | **0.00** |  |  |  |
| Road_D:sin(suntime) | -0.01 | 0.03 | -0.18 | 0.85 |  |  |  |
| Road_D:cos(suntime) | -0.04 | 0.04 | -1.22 | 0.22 |  |  |  |
| **Road_D:sin(2*suntime)** | **0.08** | **0.03** | **2.70** | **0.01** |  |  |  |
| Road_D:cos(2*suntime) | 0.05 | 0.03 | 1.63 | 0.10 |  |  |  |
| Road_D:huntingBear | -0.01 | 0.03 | -0.34 | 0.73 |  |  |  |
| sin(suntime):huntingBear | -0.07 | 0.05 | -1.28 | 0.20 |  |  |  |
| cos(suntime):huntingBear | 0.00 | 0.06 | 0.05 | 0.96 |  |  |  |
| **sin(2*suntime):huntingBear** | **-0.13** | **0.05** | **-2.73** | **0.01** |  |  |  |
| cos(2*suntime):huntingBear | -0.06 | 0.05 | -1.29 | 0.20 |  |  |  |
| Road_D:sin(suntime):huntingBear | -0.02 | 0.04 | -0.58 | 0.56 |  |  |  |
| Road_D:cos(suntime):huntingBear | -0.08 | 0.05 | -1.55 | 0.12 |  |  |  |
| Road_D:sin(2*suntime):huntingBear | -0.04 | 0.04 | -0.96 | 0.34 |  |  |  |
| **Road_D:cos(2*suntime):huntingBear** | **-0.10** | **0.04** | **-2.41** | **0.02** |  |  |  |
|  |  |  |  |  |  |  |  |
| *Subadult females* |  |  |  |  |  |  |  |
| **Intercept** | **4.70** | **0.04** | **125.81** | **0.00** |  | Intercept | 0.16 |
| **Road_D** | **-0.06** | **0.02** | **-2.40** | **0.02** |  | sin(suntime) | 0.21 |
| **sin(suntime)** | **-0.23** | **0.05** | **-4.67** | **0.00** |  | cos(suntime) | 0.81 |
| **cos(suntime)** | **-0.70** | **0.15** | **-4.73** | **0.00** |  | sin(2*suntime) | 0.32 |
| **sin(2*suntime)** | **0.41** | **0.06** | **6.35** | **0.00** |  | cos(2*suntime) | 0.29 |
| **cos(2*suntime)** | **-1.39** | **0.06** | **-23.30** | **0.00** |  | Residuals | 1.41 |
| huntingBear | 0.02 | 0.03 | 0.58 | 0.56 |  |  |  |
| Road_D:sin(suntime) | 0.04 | 0.03 | 1.44 | 0.15 |  |  |  |
| **Road_D:cos(suntime)** | **-0.09** | **0.04** | **-2.46** | **0.01** |  |  |  |
| Road_D:sin(2*suntime) | 0.03 | 0.03 | 0.88 | 0.38 |  |  |  |
| Road_D:cos(2*suntime) | -0.01 | 0.03 | -0.45 | 0.65 |  |  |  |
| Road_D:huntingBear | 0.04 | 0.03 | 1.32 | 0.19 |  |  |  |
| sin(suntime):huntingBear | 0.02 | 0.05 | 0.48 | 0.63 |  |  |  |
| cos(suntime):huntingBear | -0.01 | 0.05 | -0.21 | 0.83 |  |  |  |
| sin(2*suntime):huntingBear | -0.05 | 0.04 | -1.06 | 0.29 |  |  |  |
| cos(2*suntime):huntingBear | 0.08 | 0.04 | 1.78 | 0.07 |  |  |  |
| **Table S1 (Continued)** |  |  |  |  |  |  |  |
|  |  |  |  |  |  |  |  |
| Road_D:sin(suntime):huntingBear | -0.04 | 0.04 | -0.97 | 0.33 |  |  |  |
| Road_D:cos(suntime):huntingBear | 0.03 | 0.05 | 0.60 | 0.55 |  |  |  |
| Road_D:sin(2*suntime):huntingBear | 0.03 | 0.04 | 0.80 | 0.42 |  |  |  |
| Road_D:cos(2*suntime):huntingBear | 0.01 | 0.04 | 0.21 | 0.83 |  |  |  |
|  |  |  |  |  |  |  |  |
| *Males* |  |  |  |  |  |  |  |
| **Intercept** | **4.54** | **0.16** | **29.00** | **0.00** |  | Intercept | 0.42 |
| Road_D | -0.07 | 0.06 | -1.17 | 0.24 |  | Residuals | 1.73 |
| **sin(suntime)** | **-0.34** | **0.09** | **-3.84** | **0.00** |  |  |  |
| **cos(suntime)** | **0.86** | **0.09** | **9.06** | **0.00** |  |  |  |
| sin(2*suntime) | 0.07 | 0.08 | 0.85 | 0.39 |  |  |  |
| **cos(2*suntime)** | **-1.25** | **0.08** | **-16.34** | **0.00** |  |  |  |
| huntingBear | -0.05 | 0.12 | -0.39 | 0.69 |  |  |  |
| Road_D:sin(suntime) | -0.10 | 0.07 | -1.46 | 0.14 |  |  |  |
| Road_D:cos(suntime) | -0.07 | 0.08 | -0.88 | 0.38 |  |  |  |
| Road_D:sin(2*suntime) | -0.06 | 0.07 | -0.87 | 0.38 |  |  |  |
| Road_D:cos(2*suntime) | -0.02 | 0.07 | -0.21 | 0.83 |  |  |  |
| Road_D:huntingBear | 0.00 | 0.09 | -0.03 | 0.98 |  |  |  |
| sin(suntime):huntingBear | -0.10 | 0.14 | -0.67 | 0.50 |  |  |  |
| cos(suntime):huntingBear | -0.06 | 0.15 | -0.41 | 0.68 |  |  |  |
| sin(2*suntime):huntingBear | 0.07 | 0.12 | 0.60 | 0.55 |  |  |  |
| cos(2*suntime):huntingBear | 0.00 | 0.12 | -0.02 | 0.99 |  |  |  |
| Road_D:sin(suntime):huntingBear | -0.09 | 0.11 | -0.85 | 0.40 |  |  |  |
| Road_D:cos(suntime):huntingBear | -0.22 | 0.13 | -1.68 | 0.09 |  |  |  |
| Road_D:sin(2*suntime):huntingBear | -0.06 | 0.11 | -0.51 | 0.61 |  |  |  |
| Road_D:cos(2*suntime):huntingBear | -0.08 | 0.12 | -0.71 | 0.48 |  |  |  |

Note: Road_D stands for distance to the closest road (m).

**
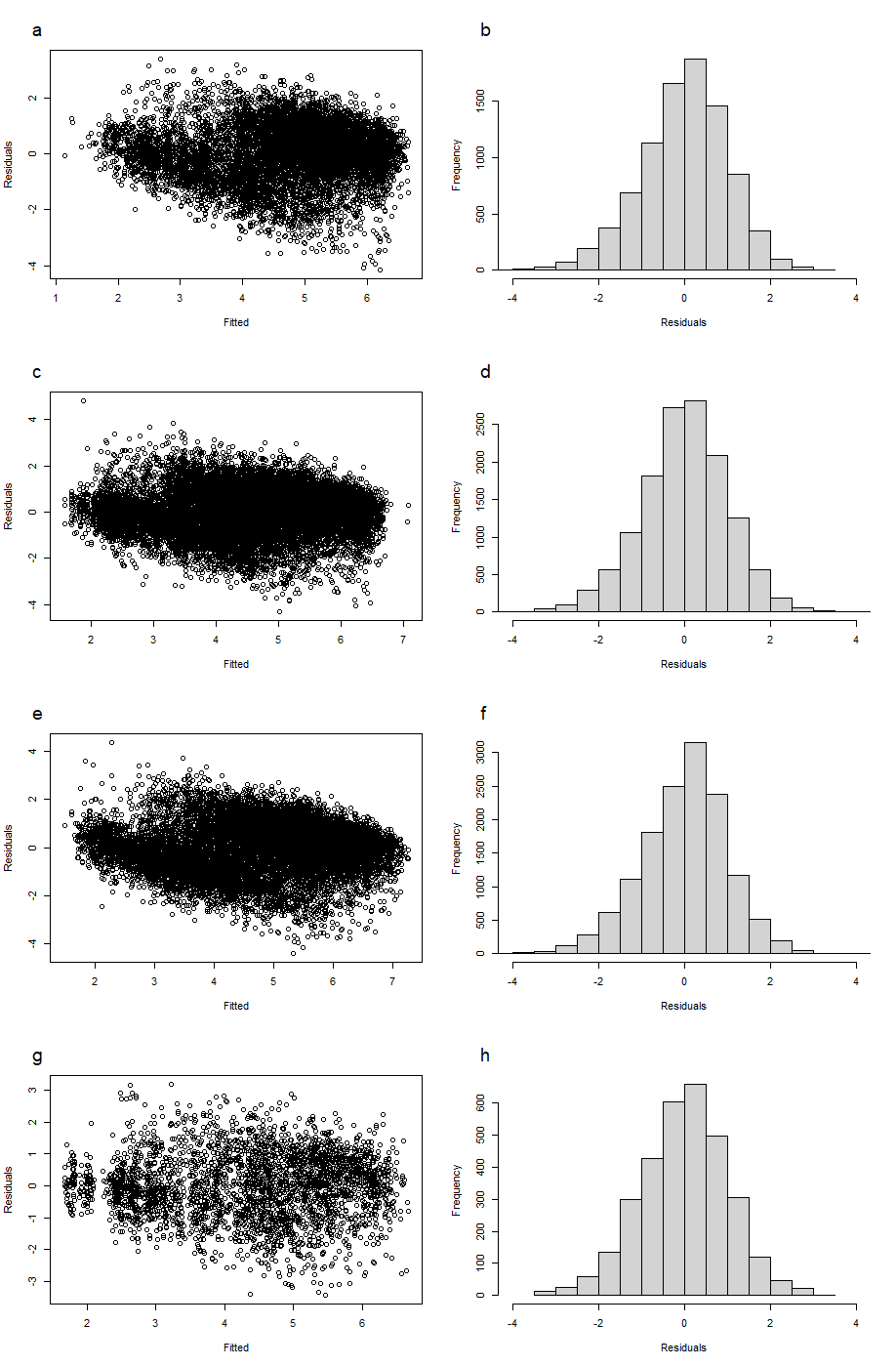
**

**Figure S1**. Diagnostic plots used to evaluate the assumptions of homoscedasticity and normality of residuals in linear models for the movement rate of brown bears. The Fitted vs Residuals relationship is presented in the left column, whereas the right column shows the distribution of residuals with histograms. Panels a and b are for females with dependent offspring, panels c and d are for lone females, panels e and f are for subadult females, whereas panels g and h are for males.
